# 331. Blood Culture Identification (BCID) Performance in Polymicrobial Bacteremia

**DOI:** 10.1093/ofid/ofac492.409

**Published:** 2022-12-15

**Authors:** William Bradford, Monica Donnelley, Jeffrey Fine, Scott Crabtree

**Affiliations:** University of California Davis Medical Center, Sacramento, California; University of California Davis Medical Center, Sacramento, California; University of California, Davis, Davis, California; University of California Davis Medical Center, Sacramento, California

## Abstract

**Background:**

The rapid multiplex PCR (rmPCR)-based FilmArray® blood culture identification (BCID) assay reduces time from positive blood culture to organism identification. Polymicrobial bacteremia is a known area of reduced diagnostic fidelity for BCID and remains incompletely characterized.

**Methods:**

All cases of clinically confirmed polymicrobial bacteremia at a large academic single center from a 23-month period were evaluated in a retrospective cohort analysis (figure 1). Samples were assorted into BCID/blood culture concordant and BCID/blood culture discordant groups. Clinical characteristics of the two groups were compared, missed organisms were characterized, and changes in antimicrobial regimen in response to BCID results were characterized.

**Figure 1.**
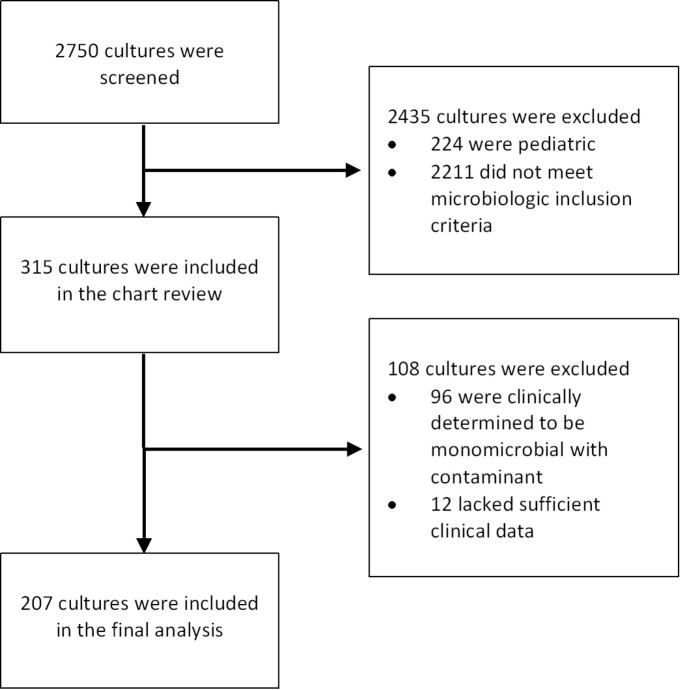

Screening and exclusion process. 207 cultures were included in final analysis from a number screened of 2750 (constituting all positive blood cultures over a 23-month period from February 2019 to January 2021). Microbiologic inclusion criteria were as follows: evidence on final phenotypic culture of at least two separate microorganisms from the same blood culture specimen as long as both organisms were species other than coagulase negative staphylococci.

**Results:**

A total of 207 samples were identified and studied. Overall, 49.3% (N=102) of polymicrobial cultures were incompletely identified by FilmArray® result. There were no significant group differences in comorbidity status, length of stay, mortality, or source between patients with polymicrobial bacteremia who had complete versus incomplete BCID identification (see table 1). Some 29.9% (38 of 127 total) of species identified corresponded to an organism potentially requiring time-sensitive treatment (relative numbers of each shown in table 2). De-escalation from adequate empiric to inadequate step-down antibiotic coverage following incomplete BCID result occurred in only 8.8% (N=9) of cases (shown in table 3).

**Table 1.**
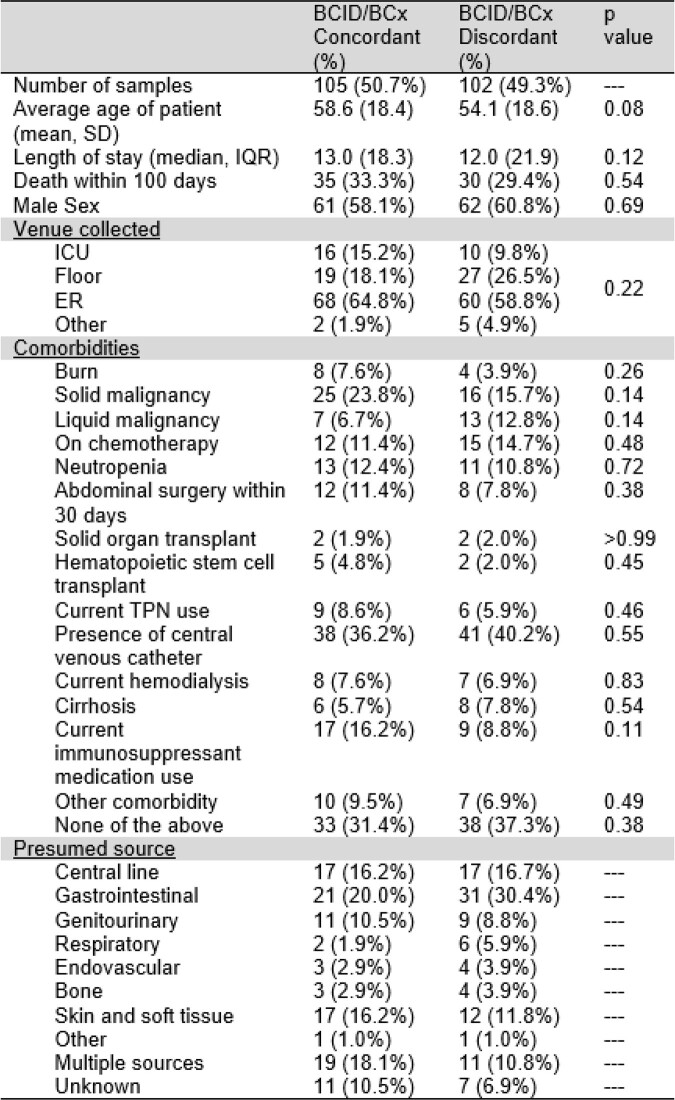

Comparison of the characteristics of the BCID/blood culture (BCx) concordant and BCID/BCx discordant groups. Abbreviations: BCx, blood culture; IQR, interquartile range; ICU, intensive care unit; ER, emergency room; TPN, total parenteral nutrition.

**Table 2.**
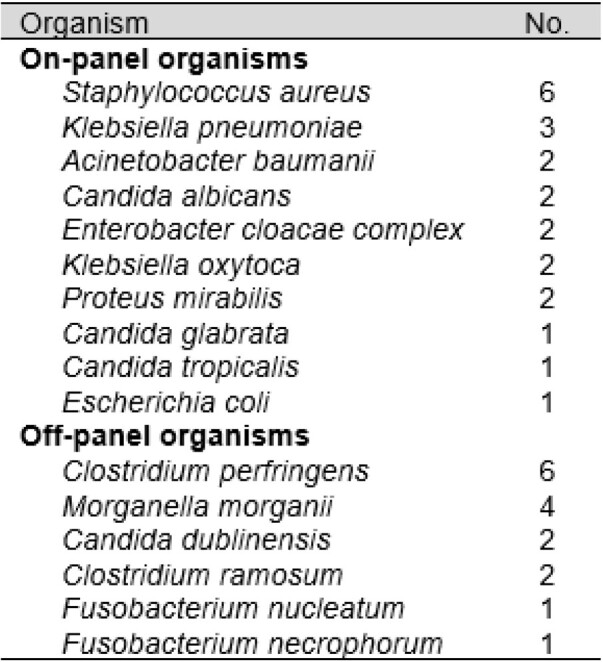

Discrepant organisms typically requiring timely treatment identified on blood culture phenotyping but not on BCID. A complete list can be found in supplemental table S1.

**Table 3.**
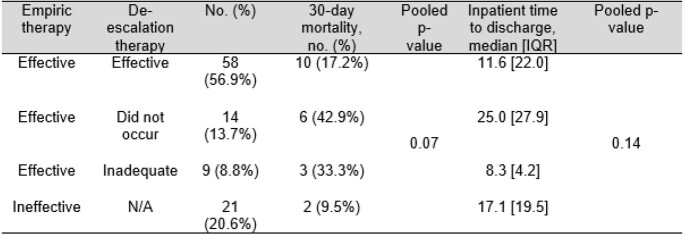

Among patients with inaccurate BCIDs, antimicrobial changes and outcomes following return of BCID result are shown below. P-values comparing the four groups are shown. There was no significant association that could be seen in mortality and time to discharge and patient’s empiric therapy and de-escalation therapy status. Abbreviations: IQR, interquartile range; N/A, not applicable.

**Conclusion:**

BCID frequently results in incomplete identification of blood culture results in patients with polymicrobial bacteremia, but clinical characteristics and outcomes were similar to those of patients with accurate BCID identification. Clinical team de-escalation to inappropriate antibiotic coverage following return the BCID assay was uncommon and was not clearly associated with inferior outcomes.

**Disclosures:**

**All Authors**: No reported disclosures.

